# Comparative performance of multiple predictive models constructed for budesonide nebulized formulation requirements

**DOI:** 10.3389/fphar.2026.1937185

**Published:** 2026-09-29

**Authors:** Jian Chen, Xihui Yu, Ting Li, Shanglian Shen, Runmin Guo, Jingcheng He, Zhouyan Wang, Jinjin Chen, Weiqiang Zeng

**Affiliations:** 1 Shunde Women and Children’s Hospital of Guangdong Medical University, Shunde, China; 2 The Second Affiliated Hospital of Shantou University Medical College, Shantou, China; 3 The Eighth Affiliated Hospital of Southern Medical University (The First People’s Hospital of Shunde District), Foshan, China

**Keywords:** budesonide nebulized formulation, demand, performance evaluation, predictive models, time series

## Abstract

**Background:**

Developing rational demand forecasting plans for National Centralized Volume-Based Procurement (NCVBP) pharmaceuticals is critical to ensuring patient access while avoiding drug inventory surplus, thereby facilitating refined hospital management and reducing operational costs. This study therefore compared the predictive performance of multiple models to identify an optimal approach for forecasting hospital demand for budesonide nebulized formulation.

**Methods:**

Using 48 months of actual utilization data (2022–2025) for budesonide nebulized formulation, a fifth-batch NCVBP product, we systematically evaluated the forecasting performance of 10 single models, 3 hybrid models, and 1 weighted ensemble model.

**Results:**

On the pediatric hospital dataset, the TBATS-XGB hybrid model achieved the best performance on the test set, yielding the lowest mean absolute percentage error (MAPE) of 12.43%, along with a root mean square error (RMSE) of 5486.0 and a mean absolute error (MAE) of 3659.8.

**Conclusion:**

The TBATS-XGB hybrid model can serve as a valuable reference for NCVBP drug quantity forecasting, addressing the limitations of conventional empirical estimation methods that often fail to capture structural changes and seasonal fluctuations in demand. Moreover, because it requires only historical usage data for deployment, this approach may be highly generalizable to hospital settings with limited data infrastructure.

## 1 Introduction

As China’s healthcare reform deepens, the zero-markup drug policy and the DRG-based payment settlement system have fundamentally altered the revenue-expenditure structure of pharmaceuticals within hospitals (Zhang et al., 2024; Du et al., 2025). Currently, most hospitals formulate drug procurement plans empirically based on historical usage, such as relying on the previous month’s consumption or triggering reorder points based on preset thresholds. These conventional procurement approaches often result in mismatches between planned procurement volumes and actual demand in subsequent periods (İşli and Aydın, 2026). Even in hospitals that have adopted intelligent supply chain systems, accurate demand forecasting remains elusive (Shen et al., 2024). Since the implementation of the NCVBP policy in 2019, substantial price reductions have been achieved, albeit accompanied by a series of challenges (Chang et al., 2024; Zhu et al., 2023; Xing et al., 2022). From the hospital perspective, ensuring an uninterrupted drug supply remains the foremost priority. When submitting procurement quantity requests under NCVBP, most hospitals currently base their estimates on the previous year’s historical purchase volumes, scaled by a certain proportion (Qiu et al., 2025), which introduces a lag in responding to demand fluctuations and may result in inaccurate reported quantities (Wang et al., 2024). Overestimation of drug demand leads to excessive procurement and inventory buildup, thereby increasing the risk of drug expiration and wastage; conversely, underestimation may precipitate supply shortages and stockout risks (Dong et al., 2023; Aronson, 2025). Furthermore, inaccurate reporting quantities may incentivize hospitals to resort to clinically inappropriate prescribing practices to fulfill procurement targets or, alternatively, to shift prescribing patterns to avoid such targets, thereby compounding the complexity of inventory management.

Accurate drug demand forecasting is a critical component for ensuring pharmaceutical supply in healthcare facilities, reducing inventory costs, and minimizing waste. Traditional forecasting models primarily include autoregressive integrated moving average (ARIMA) and exponential smoothing methods. Although these approaches exhibit limited performance when handling highly complex data, they remain popular choices due to their simplicity and transparency (He et al., 2025). In recent years, with the growing scale of medical data, machine learning and deep learning methods have increasingly become important means of improving forecasting accuracy (Mbonyinshuti et al., 2022; Rathipriya et al., 2023). To further enhance predictive capability, researchers have begun exploring hybrid/integrated modeling frameworks that combine statistical models with machine learning to balance interpretability and nonlinear fitting capacity (Yahya et al., 2026). Although existing studies have made progress in retail markets, epidemic surveillance, and large-scale supply chain settings, research on fine-grained forecasting specifically at the hospital level—particularly in low- and middle-income countries—remains relatively insufficient (Samniang et al., 2026). Hospital pharmaceutical demand data are often characterized by limited sample sizes, low (monthly) temporal frequency, and substantial noise; consequently, systematic research on demand forecasting for such data warrants further investigation.

When constructing a hospital-level demand forecasting model for pharmaceutical products, it is essential to balance predictive accuracy with interpretability, often necessitating iterative refinement and optimization to identify the most robust model. To address this challenge, we conducted an exploratory study at a children’s hospital, selecting budesonide nebulized preparations—included in China’s fifth batch of National Centralized Volume-Based Procurement (NCVBP)—as the target drug. We developed forecasting models using traditional time-series methods, machine learning algorithms, and hybrid modeling approaches, and systematically compared their predictive performance. For the first time, we systematically compared 10 single models, 3 hybrid models, and 1 weighted ensemble model on the same dataset of hospital pharmaceutical demand. This study aims to explore and establish an effective forecasting framework for budesonide nebulized preparation demand at the hospital level under the NCVBP paradigm in China.

## 2 Materials and methods

### 2.1 Data source and preprocessing

The time-series data used in this study were derived from the monthly consumption (in mg) of nebulized budesonide formulations at a children’s hospital in Foshan, China, covering the period from January 2022 to December 2025, for a total of 48 months with a monthly frequency. The data were directly extracted from the operations management module of the hospital information system and included only three fields: drug name, drug specification, and monthly consumption quantity. No patient-level information (e.g., names, medical record numbers, or diagnostic data) was involved, nor did the study involve any human tissue samples or animal experiment data.

Check for missing values in the data. If any are present, impute them using linear interpolation via the na. interp function. Identify outliers using the interquartile range (IQR) method. For detected outliers located in the interior of the series, replace them with the arithmetic mean of the two adjacent observations; for outliers at the series endpoints, substitute the sole neighboring observation, while retaining the original values for subsequent sensitivity analyses. Partition the complete time series into a training set (January 2022–December 2024, 36 months) and a test set (January 2025–December 2025, 12 months). The training set is used for model fitting and parameter estimation, and the test set is reserved for final performance evaluation. All models are compared under the identical training–test split.

### 2.2 Stationarity testing and time series feature analysis

Stationarity Testing: The KPSS test and the ADF test are employed to assess the stationarity of both the original series and the differenced series. The KPSS trend test is applied to the original series, while the KPSS level test is applied to the differenced series. Based on the test results, the non-seasonal differencing order *d* and the seasonal differencing order *D* are determined—specifically, the OCSB test is used to determine *D*, and the KPSS level test is used to determine *d*.

STL Decomposition: The training series is decomposed into seasonal, trend, and residual components using the LOESS (locally estimated scatterplot smoothing)-based seasonal-trend decomposition procedure. The seasonal window is set to the period (12 months), and robust iterative estimation is employed.

Autocorrelation Analysis: The autocorrelation function (ACF) and partial autocorrelation function (PACF) plots are generated for both the original series and the differenced series, with the number of lags set to 40 and 95% confidence intervals overlaid.

### 2.3 Development of predictive models

A total of ten single models, three hybrid models, and one weighted ensemble model were developed, encompassing classical time series models, machine learning models, deep learning models, and their hybrid variants. A fixed random seed (123) was applied to all stochastic processes to ensure reproducibility. All models were fitted on the training set (36 months) and subsequently used for extrapolative forecasting on the test set (12 months).

#### 2.3.1 Classical time series models

Seasonal naive (Snaive): The last complete seasonal cycle (12 months) of observations is used as the forecast for each future period.

ARIMA: The optimal non-seasonal ARIMA model was selected using the automatic ARIMA procedure (auto.arima) based on the AICc criterion, with the differencing order d searched over the range 0–2 and no seasonal component included.

SARIMA: A seasonal ARIMA model was constructed by incorporating a seasonal component with a period of 12 into the ARIMA framework; the seasonal differencing order D and both non-seasonal and seasonal AR/MA orders were automatically searched.

ETS: The exponential smoothing model was automatically chosen among additive/multiplicative error, trend, and seasonal components, with selection based on the AICc.

ETS_AAA: An exponential smoothing model with additive errors, additive trend, and additive seasonality was specified (i.e., forced).

TBATS_Optim: The TBATS model—which features trigonometric seasonality, Box-Cox transformation, ARMA errors, trend, and seasonal periods—was applied, with all components automatically optimized.

Holt_Winters additive model: Holt–Winters exponential smoothing with a fixed additive seasonal component.

Holt_Winters multiplicative model: Holt–Winters exponential smoothing with a fixed multiplicative seasonal component.

#### 2.3.2 Machine learning models

XGBoost: The gradient boosting tree framework was employed, with features comprising lag-1, lag-2, and lag-12 terms, as well as 11 monthly dummy variables (with December as the reference). Hyperparameter optimization was performed via grid search over the entire training data, encompassing 144 parameter combinations. The search space was defined as: eta ∈ {0.01, 0.05, 0.1, 0.2}, max_depth ∈ {3, 5, 7, 9}, subsample ∈ {0.7, 0.8, 0.9}, and colsample_bytree ∈ {0.7, 0.8, 0.9}. The regularization parameters lambda and alpha were fixed at 1 and 0.5, respectively, and were not included in the search. A 5-fold cross-validation procedure was adopted, with an early stopping round of 30, and the optimal parameters were selected by minimizing the root-mean-square error (RMSE) on the validation set. The final optimal parameters were then fixed and used for all training windows (including cross-validation and test-set predictions) without further re-tuning.

LSTM: Based on preliminary experiments, a single-layer LSTM network was constructed with 16 hidden units, dropout and recurrent dropout rates both set to 0.2, and L2 regularization (coefficient = 0.001). The input sequence length was 12 (i.e., lag-12 terms). The model was trained using the Adam optimizer with a learning rate of 0.001 and mean squared error as the loss function. The training procedure allowed up to 80 epochs with early stopping of 10 epochs based on training loss, and the best-performing weights were restored. All input data were standardized prior to training (subtracting the mean and dividing by the standard deviation), and the predictions were inversely transformed to the original scale. This architecture was fixed and no hyperparameter search was performed.

#### 2.3.3 Hybrid models

ARIMA-LSTM: An optimal non-seasonal ARIMA model was first fitted to the training series, and the residuals were extracted. These residuals were then standardized (by subtracting the mean and dividing by the standard deviation) and modeled using an LSTM network. The LSTM architecture was fixed with 16 hidden units, dropout and recurrent dropout rates both set to 0.2, no L2 regularization applied, and an input sequence length (lookback) of 12. The model was trained with the Adam optimizer (learning rate = 0.001) using mean squared error as the loss function, for up to 80 epochs with early stopping of 10 epochs based on training loss. The residual predictions were inversely transformed to the original scale. The final forecast was obtained as the sum of the ARIMA forecast and the LSTM residual forecast.

SARIMA-LSTM: Similarly, an optimal SARIMA model (with a seasonal period of 12) was fitted to the training series, and the residuals were extracted and modeled using the identical LSTM architecture described above. The final forecast was the sum of the SARIMA forecast and the LSTM residual forecast.

TBATS-XGB: A TBATS model was first fitted to the training series with the following settings: use. arma = TRUE, use. trend = TRUE, seasonal. periods = 12, and Box–Cox transformation allowed. The fitted values were obtained and the residuals were extracted. Subsequently, XGBoost was applied to model the residual series, using features that included lag-1, lag-2, and lag-12 terms, as well as 11 monthly dummy variables (with December as the reference). All XGBoost hyperparameters except the number of boosting rounds (nrounds) were pre-fixed as follows: eta = 0.05, max_depth = 3, subsample = 0.8, colsample_bytree = 0.8, lambda = 1, alpha = 0.5, and the objective function set to reg:squarederror. Only nrounds was determined via 5-fold cross-validation on the training residuals, based on minimizing the RMSE on the validation set, without performing a grid search for other hyperparameters. The final forecast was the sum of the TBATS forecast and the XGBoost residual forecast.

#### 2.3.4 Weighted ensemble model

A weighted ensemble model, denoted as Weighted_Ensemble, was constructed comprising three base models: SARIMA, TBATS_Optim, and XGBoost. The ensemble weights were determined based on the mean absolute percentage error (MAPE) of each base model evaluated via a 6-step rolling-window cross-validation procedure, with an initial training length of 24 months, a step size of 3 months, and a forecast horizon of 6 months, yielding a total of 3 folds. Specifically, the reciprocal of the MAPE for each model was computed as the initial weight, and these weights were then normalized so that the three weights summed to unity. These weights were pre-computed on the training set and fixed thereafter, and subsequently applied to the test-set predictions. The final ensemble forecast was obtained as the weighted linear combination of the forecasts from the three constituent models.

### 2.4 Model evaluation and validation strategy

#### 2.4.1 Two cross-validation schemes for predictive stability assessment

Two cross-validation schemes are employed to evaluate the predictive stability of the models.

Six-step rolling window cross-validation: The initial training set spans 24 months. At each iteration, the training window is advanced by 3 months, and predictions are generated for the subsequent 6 months. This procedure is repeated until a full 6-step ahead forecast can no longer be completed, yielding three folds in total. For each fold, performance metrics are computed over all point-wise predictions against their corresponding ground-truth values.

Twelve-step single-validation: The model is fitted using the first 24 months of the training set and then used to forecast the next 12 months (i.e., months 25 through 36 of the original training period). This scheme mimics medium-term predictive capability under a fixed training window length.

For both validation schemes, all models are evaluated with the same forecast horizon (*h* = 6 or 12, as applicable). Machine learning models (XGBoost, LSTM, and the hybrid model) retain identical hyperparameters across each training window, without re-tuning. The weighted ensemble model is excluded from cross-validation; its weights are derived from the 6-step CV results and are applied solely to the test set, thereby avoiding the use of the same data for both weight determination and performance evaluation.

#### 2.4.2 Performance metrics and model selection

For both the test-set forecast (12-month horizon) and the cross-validation predictions, the following metrics are calculated: root mean squared error (RMSE), mean absolute error (MAE), and mean absolute percentage error (MAPE). The optimal forecasting model is selected as the one that minimizes MAPE on the test set.

### 2.5 Statistical inference and robustness checks

#### 2.5.1 Permutation test

If the best model on the test set belongs to the LSTM family (i.e., LSTM, ARIMA-LSTM, or SARIMA-LSTM), a permutation test is conducted to evaluate the statistical significance of the predictions. The procedure is as follows: the forecasted values are held fixed, while the actual test-set observations are randomly permuted without replacement 500 times. For each permutation, the MAPE is recalculated. The *p*-value is then defined as the percentile of the original MAPE within the empirical permutation distribution. The null hypothesis states that the model’s predictions are indistinguishable from random guessing (i.e., the prediction error is no smaller than that obtained from randomly permuted series).

#### 2.5.2 Block bootstrap robustness check

To assess the uncertainty of prediction errors for the best-performing model on the test set, a block bootstrap resampling procedure is implemented. The block length is set to 12 (corresponding to one seasonal cycle), with B = 200 resampling iterations. In each iteration, contiguous blocks of length 12 are drawn with replacement from the training set and concatenated to form a pseudo-sample of the same length as the original training set. The model is refitted on each pseudo-sample, predictions are generated for the test set, and both RMSE and MAPE are computed. This yields empirical distributions of 200 RMSE and MAPE values, from which 95% percentile confidence intervals are extracted.

#### 2.5.3 Outlier sensitivity analysis

To evaluate the impact of outlier correction on model performance, the original data with outliers retained (i.e., uncorrected) are used. The training set (2022–2024) and test set (2025) are re-partitioned, and the SARIMA model is refit to compute the test-set MAPE. This MAPE is then compared with that obtained from the SARIMA model fitted on the outlier-corrected data, thereby quantifying the sensitivity of the results to the outlier treatment.

### 2.6 Prediction intervals and coverage rates

For classical models (SARIMA, ARIMA, ETS, and TBATS_Optim), prediction intervals are constructed using the models’ inherent methods, which are based on the assumption of normally distributed residuals or the model structure. Intervals are computed at both the 80% and 95% nominal confidence levels.

For all other models, including the proposed best model, prediction intervals are generated via the residual bootstrap method. Specifically, using the best model as an example, a residual sequence of length equal to the test set size is drawn with replacement from the training-set fitted residuals (or differenced residuals). This bootstrapped residual sequence is then added to the point forecasts. The procedure is repeated 500 times to obtain an empirical distribution of the forecast values, from which the 80% and 95% percentile intervals are derived.

Coverage rates are calculated as the proportion of the 12 actual test-set observations that fall within the computed prediction intervals. These empirical coverage rates are then compared with the nominal confidence levels of 0.80 and 0.95.

### 2.7 Monthly error pattern analysis

For all models, the absolute percentage errors (APE) on the test set were aggregated by calendar month. For each month, the mean, median, and standard deviation of APE were computed to assess the temporal dependence of prediction errors.

### 2.8 Software and computing environment

All statistical analyses were performed using R version 4.4.1, running on Windows 11 x64. The primary R packages employed included: forecast (9.0.0) for classical time series models, xgboost (3.2.1) for gradient boosting trees, keras3 (1.5.0) and tensorflow (2.20.0) for LSTM and its hybrid variants, ggplot2 (4.0.1) for visualization, and tseries (0.10-59) and DescTools (0.99.60) for stationarity testing and descriptive statistics. A random seed of 123 was uniformly set to ensure reproducibility. LSTM computations were performed using Python 3.11 and invoked via the reticulate package in R.

## 3 Results

### 3.1 Series characteristics and stationarity

The original series comprised 48 consecutive monthly observations from January 2022 to December 2025. Descriptive statistics indicated a mean of 29,340.0 (unit: mg), a standard deviation of 9,188.6, a minimum of 9,578, a maximum of 47,926, a skewness of 0.109, and a kurtosis of −0.351. The time plot of the original series is presented in Supplementary Figure S1.

Stationarity was assessed using the KPSS and ADF tests. For the original series, the KPSS trend test statistic was 0.121 *(p* = 0.096), and the ADF test statistic was −2.258 (*p* = 0.473), indicating non-stationarity. A non-seasonal differencing order of *d* = 1 and a seasonal differencing order of *D* = 0 were suggested. After first differencing, the KPSS level test statistic was 0.056 (*p* = 0.1), and the ADF test statistic was −5.499 (*p* = 0.01), confirming that the differenced series achieved stationarity. The ACF and PACF plots for both the original and differenced series are shown in Supplementary Figures S2–S5.

### 3.2 Time series decomposition and model goodness-of-fit

STL decomposition (with a seasonal period of 12 and robust LOESS) was applied to the training series (2022–2024), with results presented in Supplementary Tables S1, S2. The variances of the seasonal, trend, and residual components accounted for 71.1%, 13.4%, and 42.6% of the total variance, respectively (the sum of the component variances exceeded the total variance owing to the non-orthogonal nature of the decomposition). Plots of the seasonal, trend, and residual components are provided in Supplementary Figures S6–S8.

The AIC and BIC values for the SARIMA, TBATS, and ETS models fitted to the training set are reported in Supplementary Tables S1, S3. The SARIMA model yielded the lowest AIC (473.8) and BIC (476.1). The TBATS model structure does not directly provide a BIC value.

### 3.3 Cross-validation model comparison

To evaluate the predictive stability of each model, a 6-step rolling-window cross-validation (with 3 folds) and a 12-step single-validation (with 1 fold) were employed.

#### 3.3.1 6-Step rolling-window CV

The performance metrics (RMSE, MAE, MAPE) for each model are presented in Table 1. In ascending order of MAPE, the top five models were XGBoost (21.46%), Snaive (25.69%), ARIMA (27.27%), ETS (28.34%), and ARIMA-LSTM (30.36%), while the poorest performance was observed for HW_Multiplicative (127.67%).

**TABLE 1:** Results of the 6-step rolling-window cross-validation (3 folds).

Model	RMSE	MAE	MAPE (%)
XGBoost	7284.9	5537.8	21.46
Snaive	10706.8	7808.7	25.69
ARIMA	10055.4	7554.3	27.27
ETS	10034.4	7899.9	28.34
ARIMA-LSTM	10747.8	8355.3	30.36
SARIMA	10750.3	8776.1	31.09
SARIMA-LSTM	11306.9	9418.2	33.31
ETS_AAA	16144.8	11288.3	36.07
LSTM	15002.0	12173.1	43.00
TBATS_Optim	16917.5	14532.5	49.10
TBATS_XGB	18767.2	16317.4	54.58
HW_Additive	30242.2	19567.4	62.03
HW_Multiplicative	69025.1	42196.0	127.67

#### 3.3.2 12-Step single-validation

Using the first 24 months of the training set to forecast the subsequent 12 months (months 25–36), the results indicated that LSTM achieved the lowest MAPE (18.39%), followed by XGBoost (22.21%) and Snaive (31.64%). The poorest performance was observed for HW_Multiplicative, with a MAPE of 490.26% (Table 2).

**TABLE 2:** Results of the 12-step single-validation (1 fold).

Model	RMSE	MAE	MAPE (%)
LSTM	7376.1	6018.3	18.39
XGBoost	8039.0	5638.3	22.21
Snaive	12690.5	9840.0	31.64
ETS	17345.6	16201.3	57.72
ARIMA	17645.0	16521.6	58.78
SARIMA	17645.0	16521.6	58.78
ARIMA-LSTM	17949.9	16470.6	59.11
SARIMA-LSTM	18536.7	17510.0	62.00
ETS_AAA	29969.4	28682.6	97.89
TBATS_Optim	36652.4	32996.2	115.51
TBATS_XGB	39250.0	35984.5	123.82
HW_Additive	79243.0	72550.6	247.59
HW_Multiplicative	164797.0	146131.7	490.26

#### 3.3.3 Ranking comparison of the two validation strategies

Comparing the MAPE rankings between the 6-step CV and the 12-step validation, XGBoost ranked 1st in the 6-step CV and 2nd in the 12-step validation, whereas LSTM ranked 9th in the 6-step CV but rose to 1st in the 12-step validation.

### 3.4 Predictive performance on the test set

Using the training set from 2022 to 2024 to forecast the 12 months of 2025, the performance metrics of all models are summarized in Table 3. The TBATS_XGB model achieved the lowest MAPE (12.43%), with an RMSE of 5,486.0 and an MAE of 3,659.8, followed by TBATS_Optim (MAPE 13.06%), SARIMA (MAPE 16.11%), SARIMA-LSTM (MAPE 18.00%), and ETS (MAPE 21.02%), whereas Weighted_Ensemble yielded a MAPE of 21.79%. Plots comparing the predicted and actual values for the top-five models and Weighted_Ensemble are presented in Figures 1–6, while those for the remaining models are provided in Supplementary Figures S9–S16.

**TABLE 3:** Predictive performance of each model on the test set (January–December 2025).

Model	RMSE	MAE	MAPE (%)
TBATS_XGB	5486.0	3659.8	12.43
TBATS_Optim	5782.0	3935.4	13.06
SARIMA	5266.6	4161.6	16.11
SARIMA-LSTM	5628.4	4569.0	18.00
ETS	7126.6	5946.2	21.02
Weighted_Ensemble	6476.7	5330.1	21.79
Snaive	7945.8	6053.6	21.96
ETS_AAA	7417.9	5784.9	22.92
ARIMA	8886.2	6715.7	28.73
LSTM	8888.9	6740.2	29.22
ARIMA-LSTM	8976.8	6887.4	29.34
XGBoost	10285.5	8054.0	35.55
HW_Additive	15631.0	13564.8	53.40
HW_Multiplicative	21278.1	18315.6	66.54

**FIGURE 1:**
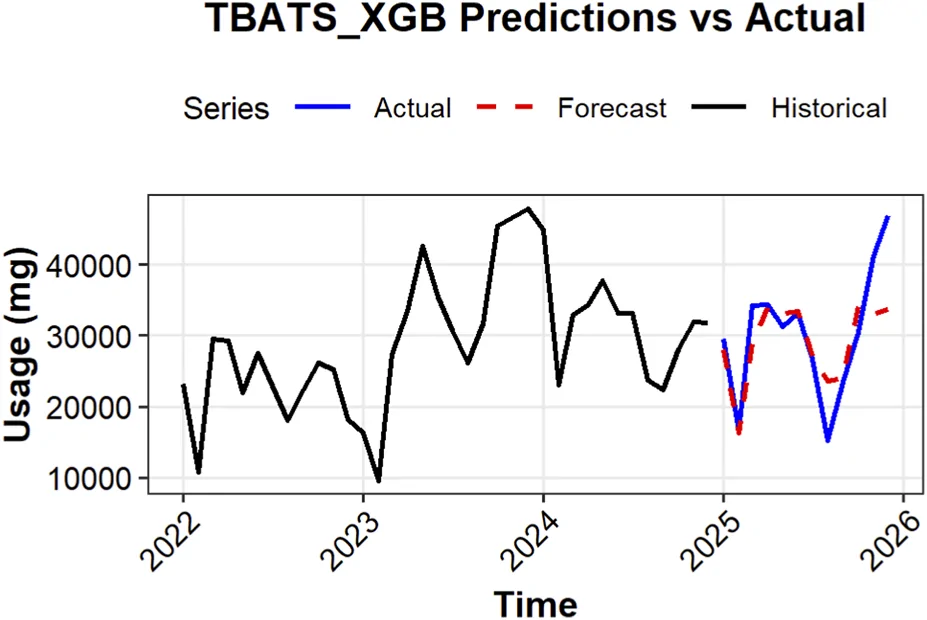
Comparison of TBATS_XGB model predictions and actual usage.

**FIGURE 2:**
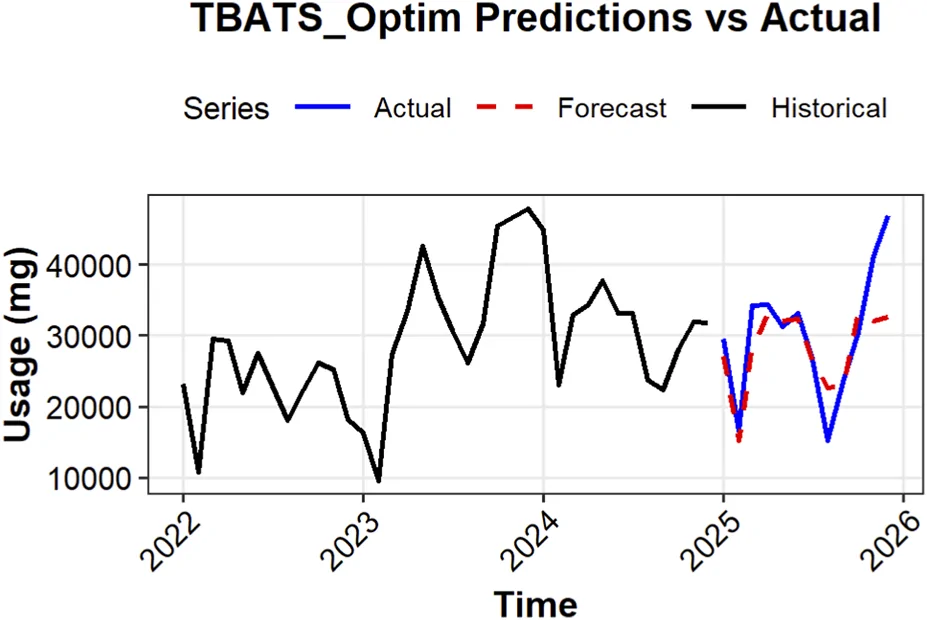
Comparison of TBATS_Optim model predictions and actual usage.

**FIGURE 3:**
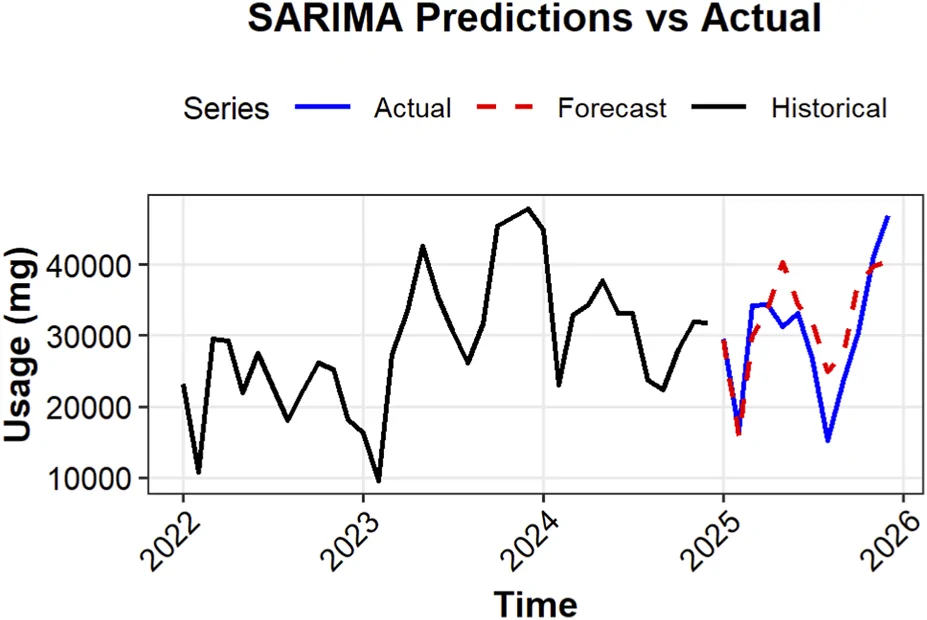
Comparison of SARIMA model predictions and actual usage.

**FIGURE 4:**
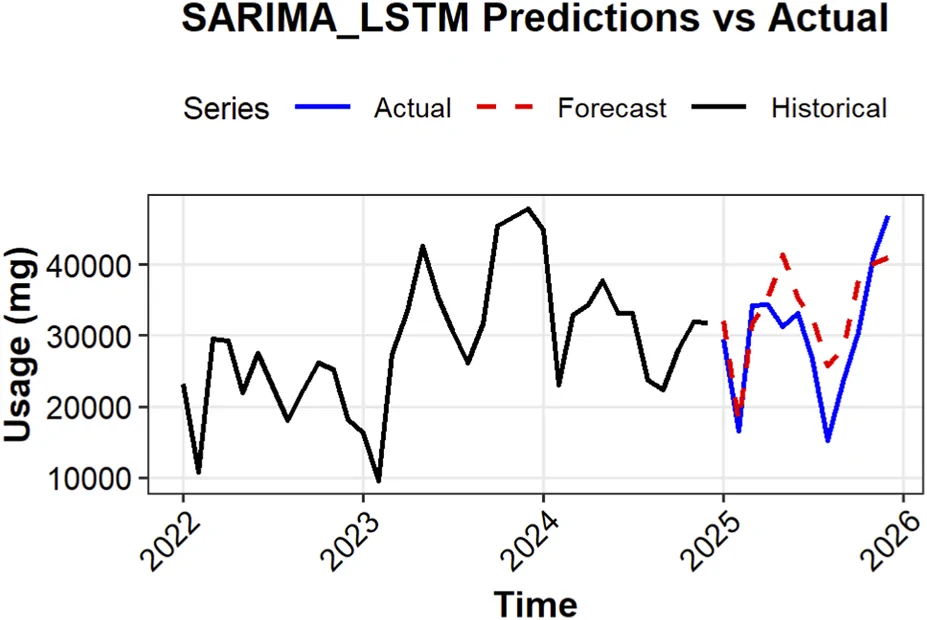
Comparison of SARIMA_LSTM model predictions and actual usage.

**FIGURE 5:**
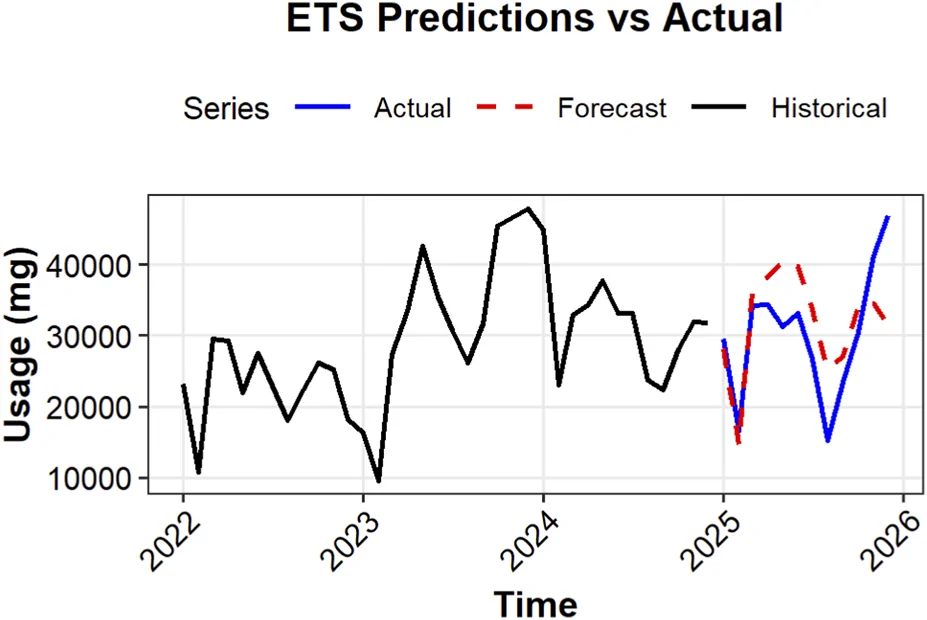
Comparison of ETS model predictions and actual usage.

**FIGURE 6:**
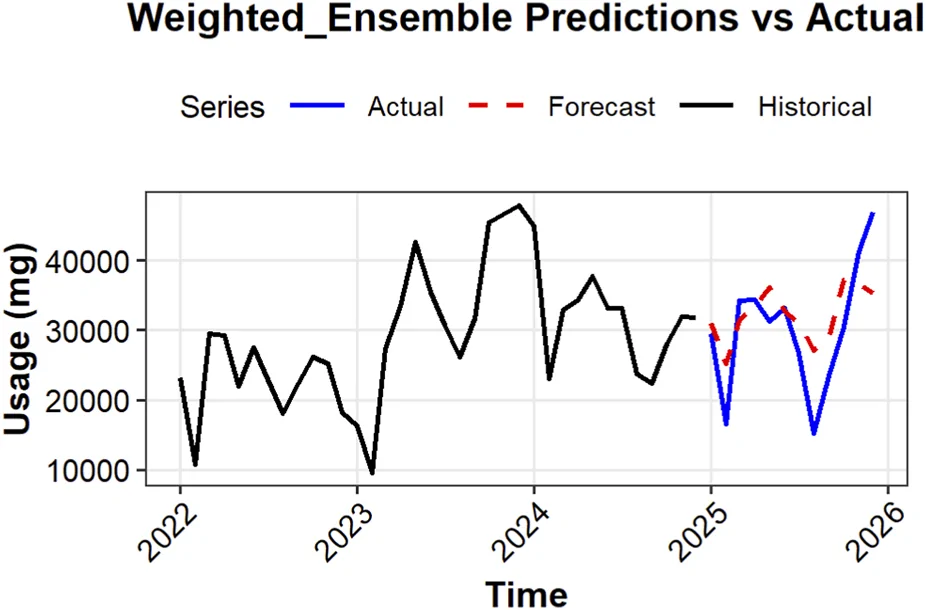
Comparison of Weighted_Ensemble model predictions and actual usage.

#### 3.4.1 Prediction interval coverage of the best model

Based on the lowest MAPE on the test set, the best-performing model was TBATS_XGB. Prediction intervals at the 80% and 95% nominal levels were constructed using the residual bootstrap method with B = 500 bootstrap replicates, where the bootstrap residuals were drawn from the training fitted residuals of TBATS_XGB. Among the 12 actual values in the test set, the actual coverage rate of the 80% prediction intervals was 83.3% (10/12), and that of the 95% prediction intervals was 100% (12/12). The prediction interval plot is shown in Figure 7.

**FIGURE 7:**
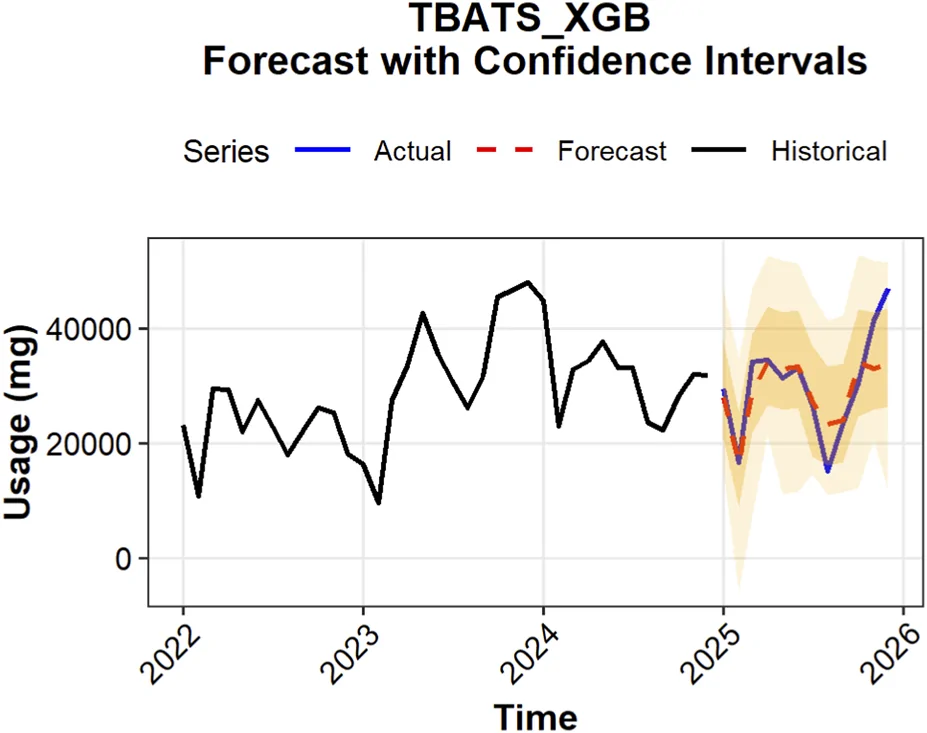
TBATS_XGB forecast with confidence intervals for usage.

#### 3.4.2 Robustness check using block bootstrap

A block bootstrap procedure with B = 200 resamples and a block length of 12 was applied to the TBATS_XGB model. The resulting 95% confidence intervals for the forecast errors were as follows: for RMSE, the 95% CI was (7,988.6, 34,621.4); for MAPE, the 95% CI was (22.6%, 105.1%). The block bootstrap prediction interval plot is shown in Figure 8.

**FIGURE 8:**
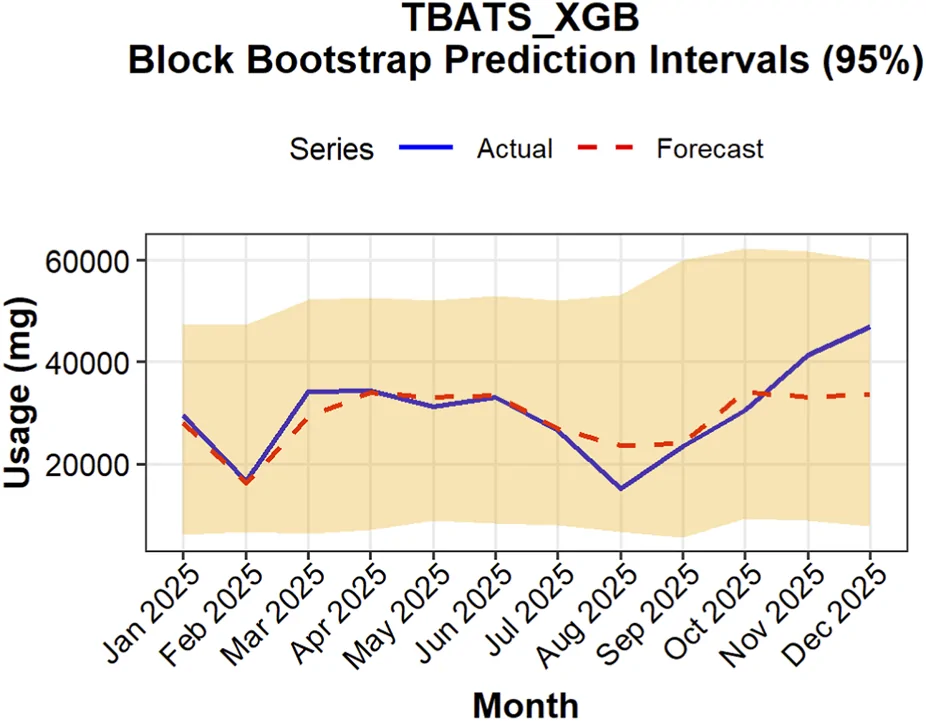
TBATS_XGB forecast with 95% bootstrap intervals for usage (2025).

### 3.5 Outlier sensitivity analysis

One outlier was detected in the original data (November 2023; original value = 52,188, corrected to 46,670.5). To assess the impact of outlier correction, the SARIMA model was fitted separately to the corrected data and to the data retaining the outlier, and the MAPE was computed on the test set. The results showed that the MAPE after outlier correction was 16.11%, whereas that with the outlier retained was 16.31%, a difference of only 0.20 percentage points.

### 3.6 Monthly forecast error analysis

The absolute percentage errors (APEs) for all models across each month of the test set were summarized, and the mean, median, and standard deviation are presented in Table 4 and Figure 9. The highest mean APE occurred in August 2025 (93.06%), followed by February (41.33%) and September (35.63%). Relatively lower errors were observed in January (9.19%), March (11.75%), April (10.54%), and June (11.17%).

**TABLE 4:** Mean absolute percentage error by month on the test set (based on monthly APEs across all models for January–December 2025).

Month	Mean APE (%)	Median APE (%)	SD APE (%)
January	9.19	5.72	12.88
February	41.33	25.63	44.03
March	11.75	8.24	8.87
April	10.54	3.62	17.43
May	27.54	17.90	32.81
June	11.17	2.93	18.54
July	26.71	22.32	22.47
August	93.06	83.31	37.93
September	35.63	29.37	33.19
October	26.16	17.73	26.58
November	17.35	19.29	9.07
December	23.92	26.67	8.58

**FIGURE 9:**
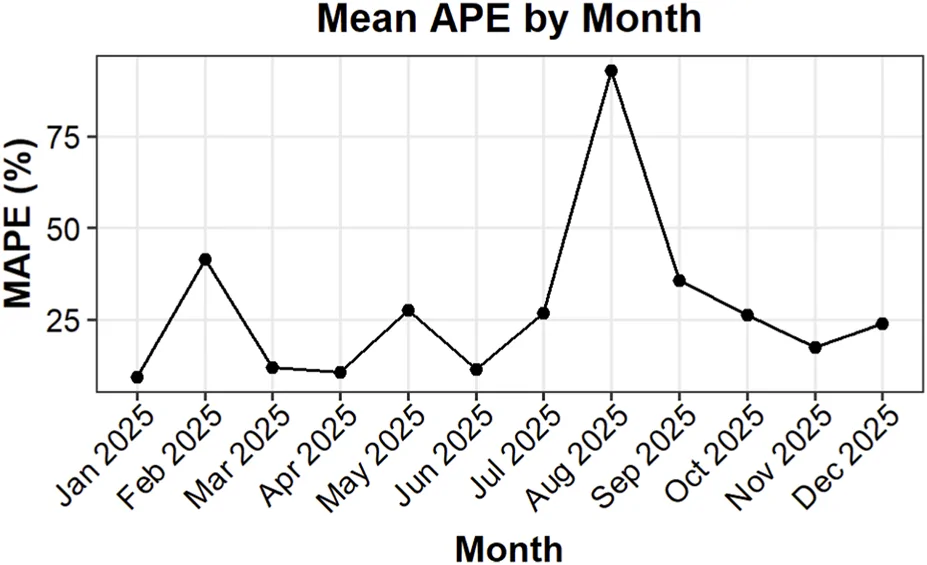
Mean Absolute Percentage Error (MAPE) by month in 2025.

## 4 Discussion

The National Centralized Volume-Based Procurement (NCVBP) represents a pivotal initiative in China’s ongoing healthcare system reform, effectively reducing drug prices through a “volume-for-price” mechanism. Under this program, medical institutions are required to submit their annual demand forecasts prior to the start of each procurement cycle. Once confirmed, these forecasts serve as the benchmark for contracted purchase volumes, directly influencing subsequent supply assurance and capital occupancy. Overestimation of demand exposes hospitals to financial strain from excess inventory and the risk of drug expiration, whereas underestimation may lead to drug shortages and disruptions in clinical supply. Compared with traditional procurement models, the demand forecasting process under NCVBP is characterized by “ex ante prediction and locked procurement,” imposing stricter requirements on forecasting accuracy and allowing considerably less margin for error. Current forecasting methods for NCVBP drugs primarily rely on empirical extrapolation from historical usage data of the preceding year, which are inadequate for capturing structural shifts in demand, seasonal fluctuations, and policy-induced shocks. In this context, developing a data-driven drug demand forecasting model is of substantial practical significance for improving the accuracy of NCVBP volume declarations and optimizing hospital pharmaceutical procurement planning. The fifth batch of NCVBP was implemented in October 2021, with budesonide nebulized formulation included as one of the procured varieties. This study collected post-implementation utilization data from medical institutions for this drug and sought to establish a robust demand forecasting model to narrow the gap between declared NCVBP volumes and actual clinical demand.

This study systematically compared the performance of 10 individual models, 3 hybrid models, and 1 weighted ensemble model in forecasting the demand for budesonide nebulized formulation. The TBATS-XGB hybrid model achieved the lowest MAPE (12.43%) on the test set, with an RMSE of 5,486.0 and an MAE of 3,659.8. Among all compared models, TBATS_Optim ranked second (MAPE = 13.06%), with a margin of 0.63 percentage points behind TBATS-XGB. The superior performance of TBATS-XGB is grounded in its two-stage architecture. In the first stage, TBATS employs Fourier series to capture seasonal patterns and applies a Box–Cox transformation to handle variance instability, thereby robustly extracting the trend and cyclical components embedded in the demand series (Bindel and Seifert, 2025). In the context of budesonide nebulized formulation—a drug with pronounced seasonal fluctuations—TBATS’s seasonal decomposition capability effectively isolates annual periodic patterns and long-term trends. However, a limitation of relying solely on TBATS is that its linear framework cannot adequately capture nonlinear fluctuations induced by exogenous events such as pandemic shocks or policy changes. This is precisely where the residual correction module of XGBoost comes into play: XGBoost performs nonlinear learning on the residuals after TBATS fitting, capturing complex fluctuation patterns that cannot be explained by the parametric components (Chen and Guestrin, 2016). This clear division of labor enables TBATS to focus on interpretable temporal structures while XGBoost handles unexplainable stochastic components, avoiding the difficulty of a single model attempting to accomplish both tasks simultaneously. Compared with TBATS_Optim, the improvement of 0.63 percentage points validates the incremental value of the residual correction module in capturing nonlinear fluctuations. This finding is consistent with existing evidence on hybrid modeling in pharmaceutical demand forecasting. Previous studies have demonstrated that separating baseline demand estimation from residual correction can significantly improve forecasting performance in drug demand scenarios prone to external disturbances (Yahya et al., 2026). Hybrid models combining metaheuristic feature optimization with gradient boosting have also achieved excellent results in hospital drug demand forecasting (Samniang et al., 2026). A weighted ensemble framework integrating ARIMA-type regression models with XGBoost has further shown that hybrid approaches enhance flexibility and accuracy under mixed demand patterns (Hammam et al., 2025). In addition, hybrid architectures that combine domain knowledge with deep learning models have confirmed the benefit of incorporating external knowledge for drug demand prediction (Chen et al., 2026). In contrast, the TBATS-XGB model adopted in this study follows a more parsimonious implementation path—it does not require the construction of external knowledge graphs and can be deployed using only historical usage data. This feature may confer better generalizability in hospital settings where data infrastructure is limited. Nevertheless, it must be acknowledged that structural breaks constitute a non-negligible challenge in pharmaceutical demand forecasting. Although the TBATS-XGB hybrid model alleviates this issue to a certain extent through the state-space adaptability of TBATS and the residual learning capability of XGBoost, its predictive performance may temporarily decline in the presence of severe and persistent structural changes, such as those arising from a novel large-scale pandemic. Should conditions permit, we recommend integrating change point detection algorithms (e.g., CUSUM or Bayesian change point detection) into practical deployment to monitor forecast errors in real time, and to trigger model recalibration when a statistically significant increase in error is detected. Furthermore, as additional data accumulate, we advise periodically retraining the model using an expanding time window (e.g., on a quarterly basis) to incorporate emerging data patterns.

This study employed two cross-validation schemes to evaluate the predictive stability of each model, revealing notable differences in generalization ability across models. XGBoost ranked first in the 6-step CV (MAPE = 21.46%) and second in the 12-step validation (MAPE = 22.21%), demonstrating satisfactory cross-temporal stability. In contrast, LSTM ranked ninth in the 6-step CV (MAPE = 43.00%) but jumped to first in the 12-step validation (MAPE = 18.39%). TBATS_Optim and TBATS-XGB performed poorly in both validation schemes, with MAPE values of 49.10% and 54.58% in the 6-step CV, and 115.51% and 123.82% in the 12-step validation, respectively, reflecting the instability of parameter estimation for time series models when the training window is short. This discrepancy can be explained by the design differences between the two validation schemes. The 6-step CV adopted a rolling window strategy, with an initial training set of 24 months, advancing by 3 months at each step to forecast the next 6 months, across three folds. In each fold, the model was independently refitted, and the training data covered only two complete seasonal cycles, subjecting LSTM to the constraint of limited samples during each refitting. By contrast, the 12-step validation also used a 24-month training set but was fitted once without multiple rounds of refitting, allowing LSTM to leverage the full seasonal cycle information and converge stably in a single fit. Thus, the instability of LSTM in the 6-step CV may stem from perturbations to parameter estimation induced by rolling refitting under small-sample conditions, whereas its superior performance in the 12-step validation suggests that its sequential learning capability can be effectively harnessed when complete seasonal cycle information is available. The 6-step CV evaluates model robustness under data-constrained conditions, while the 12-step validation assesses optimal performance on a fixed training set; these two schemes are complementary. These observations are consistent with previous findings. For instance, on limited pharmaceutical datasets, shallow neural networks have been shown to outperform deep LSTM models (Rathipriya et al., 2023). With limited data points, simpler models can effectively mitigate the risk of overfitting (Bindel and Seifert, 2025). This further supports the fundamental principle that model complexity should be aligned with data availability.

This study quantified forecast uncertainty using the residual bootstrap method (B = 500) and the block bootstrap method (B = 200, block length = 12). The actual coverage rates of the 80% and 95% prediction intervals were 83.3% and 100%, respectively, indicating that the interval estimates were well calibrated. The block bootstrap yielded a 95% CI for RMSE of [7,988.57, 34,621.39] and a 95% CI for MAPE of [22.63%, 105.12%]; the relatively wide intervals reflect the substantial uncertainty inherent in pharmaceutical demand forecasting (Rojas et al., 2021). The block bootstrap resamples the training set with replacement and refits the model for each resampled dataset, thereby capturing the impact of sampling variability in the training data on forecast outcomes (Kunsch, 1989). In contrast, the residual bootstrap reflects the predictive variability induced by residual randomness while holding model parameters fixed (Efron and Tibshirani, 1994). As these two approaches capture distinct sources of uncertainty, both should be considered in practice. Therefore, when utilizing point forecasts to inform procurement planning, it is advisable to interpret them in conjunction with interval estimates for more comprehensive decision-making.

Quantifying forecast uncertainty holds direct practical value for hospital procurement decision-making. An AI-based vendor-managed inventory (VMI) supply chain system has been shown to reduce hospital inventory levels to approximately 20% and lower the annual average inventory error rate from 0.425‰ to 0.025‰; however, it has also been noted that the absence of a scientific estimation framework can lead to significant discrepancies between procurement quantities and actual usage (Shen et al., 2024). Other research has combined forecast information with threshold adaptation based on demand volatility perception, achieving an 81.5% reduction in stockout days and a 71.6% decrease in ordering workload while maintaining an auditable requisition structure—yet a 118.9% increase in inventory exposure highlights the multifaceted trade-offs inherent in safety stock management (İşli and Aydın, 2026). The skewness and kurtosis of demand distributions have also been found to exert a significant influence on total inventory costs (Rojas et al., 2021). Inaccurate inventory quantification and erroneous demand forecasts are primary drivers of excess drug inventory (Aronson, 2025). Seasonal forecasting based on SARIMA has been shown to effectively reduce procurement-related labor costs (Shenouda et al., 2026). In light of these findings, it is advisable to integrate the interval estimation results of this study with insights from supply chain optimization research to construct a tiered decision-making framework. When the point forecast lies near the lower bound of the prediction interval, hospitals may declare the minimum required volume stipulated by the NCVBP policy (Zhang et al., 2023), while simultaneously preparing contingency procurement plans to address potential increases in demand. When the forecast approaches the upper bound of the interval, given the risk of stockouts associated with forecast uncertainty, it may be prudent to increase the declared volume or establish safety stock, while remaining cognizant of the cost trade-offs arising from elevated inventory exposure (İşli and Aydın, 2026). When the prediction interval is notably wide—as in this study, where the MAPE confidence interval spanned 82.5 percentage points—this suggests that demand for the drug may be subject to considerable uncertainty. In such cases, rather than relying on fixed procurement quantities, priority should be given to establishing flexible supplementary supply mechanisms with suppliers, enabling rapid response once actual demand becomes clearer (Shen et al., 2024; Zhang et al., 2023).

From a methodological perspective, the “baseline estimation plus residual correction” strategy offers a generalizable framework for pharmaceutical demand forecasting. Previous studies have validated the effectiveness of XGBoost in this domain, achieving an R^2^ of 0.87 on both the training and test sets in forecasting essential medicine demand in Ethiopia, significantly outperforming ARIMA (R^2^ = 0.62) (Biadigilign et al., 2026). Tree-based algorithms and neural networks are also among the most widely applied machine learning methods for predicting drug concentrations (Huang et al., 2025), indicating that such approaches possess strong applicability in pharmaceutical data analysis. From a practical standpoint, the proposed model can provide data-driven support for hospital volume declarations under the NCVBP program. Traditional empirical estimation methods are ill-suited to capturing structural shifts and seasonal fluctuations in demand (Zhang et al., 2023). Changes in the policy environment have further exacerbated this challenge; for instance, the zero-markup drug policy exerts differential effects between secondary and tertiary hospitals (Zhang et al., 2024), while Diagnosis-Related Group (DRG) payment reform significantly influences inpatient cost structures (Du et al., 2025). Moreover, external factors such as climatic conditions may serve as key determinants for improving the accuracy of drug demand forecasts (Jahani et al., 2024). One study developed a drug shortage risk prediction model incorporating predictors including NCVBP participation, therapeutic category, dosage form, distribution supplier, order timeliness, order date, and unit price, which effectively predicted the risk of drug shortages. However, that study did not address the prospective question of exactly how much volume should be declared for NCVBP varieties (Dong et al., 2023). The present study fills this gap by applying the TBATS-XGB model to prospectively forecast future demand for NCVBP-designated drugs. Together, these two lines of research enable hospitals to further quantify demand forecasts on the basis of identified shortage risk factors, thereby forming a complete decision-making chain of “risk identification → demand forecasting → precise volume declaration.”

In summary, although this study does not propose an entirely novel forecasting method, we place particular emphasis on mitigating overfitting risks under small-sample conditions through strategies such as multiple regularization and early stopping, thereby providing empirical evidence of model generalizability. We systematically validate the effectiveness of a framework that combines statistical decomposition with machine learning–based residual learning for monthly pharmaceutical demand forecasting with limited sample sizes. Furthermore, we provide a reproducible methodological framework and a set of overfitting prevention strategies, which together serve as empirical benchmarks and methodological references for future studies on pharmaceutical demand forecasting under similar data conditions.

This study has several limitations. First, the data were derived from a single tertiary hospital, comprising 48 monthly observations. Although rolling-window cross-validation and bootstrap methods partially mitigated the overfitting risk inherent in a small sample, only three folds were employed in the rolling window, which may lead to considerable variance in the evaluation estimates; hence, the results should be interpreted with caution. Therefore, the generalizability of the model to different hospital types and geographic regions remains to be validated. Second, only univariate time series data were employed, without incorporating external covariates such as respiratory disease surveillance and meteorological variables, whereas the integration of multisource data has been shown to substantially improve predictive accuracy (Li et al., 2024). Third, monthly aggregation may obscure demand fluctuations at finer temporal scales. However, for complex predictive models such as LSTM and XGBoost, we conducted a comparative analysis using weekly data. On the weekly test set, the MAPE of XGBoost was 32.36%, which was close to that on the monthly data (35.55%); in contrast, the MAPE of LSTM was 50.09%, substantially worse than its monthly-data performance (29.22%), suggesting that the long-term forecasting robustness of deep learning models under higher-frequency data warrants further investigation (Supplementary Sensitivity_Weekly_Test_tuned). Fourth, in a highly regulated market, the limited temporal horizon may not adequately capture long-term concept drift (Yahya et al., 2026).

Future research could pursue the following directions. First, integrating multisource external data, including respiratory disease surveillance and meteorological variables, could improve the predictive accuracy for seasonally sensitive pharmaceuticals (Li et al., 2024; Shenouda et al., 2026). Second, exploring weekly or daily data would allow for the capture of finer-grained demand patterns. Third, multicenter studies should be conducted to validate the generalizability of the models across hospital tiers and geographic regions (Joosse et al., 2025). Fourth, linking the forecasting models with inventory optimization algorithms could establish a closed-loop decision support system (İşli and Aydın, 2026; Shen et al., 2024). Fifth, incorporating policy variables would better reflect the dynamic regulatory environment (Zhang et al., 2023; Zhang et al., 2024; Du et al., 2025). Sixth, leveraging SHAP analysis could be considered to enhance model interpretability and support managerial decision-making (Yahya et al., 2026; Huang et al., 2025). Additionally, should the data volume expand in the future, it would be worthwhile to consider incorporating deep learning architectures such as Transformer, N-BEATS, and Informer for comparative evaluation.

## 5 Conclusion

This study evaluated the predictive performance of 10 single models, 3 hybrid models, and 1 weighted ensemble model using 48 months of actual utilization data (2022–2025) for budesonide nebulized formulations, a fifth-batch National Centralized Volume-Based Procurement (NCVBP) product. Among all candidate models, the TBATS-XGB hybrid achieved the best test-set MAPE of 12.43% on the pediatric hospital dataset. By extracting trend and seasonal components via TBATS and correcting nonlinear residual fluctuations with XGBoost, the hybrid effectively captured complex demand patterns while requiring only historical consumption data, suggesting favorable generalizability in hospital settings with limited data infrastructure. XGBoost maintained stable generalization under constrained sample sizes, whereas LSTM may realize its sequential learning advantages only when sufficient data are available. TBATS-based models exhibited unstable seasonal parameter estimates and suboptimal validation performance over short training windows. These findings imply that hospitals in early data-accumulation phases should prioritize models with lower sample-size requirements and progressively transition to more complex architectures as data accrue. Prediction intervals were well-calibrated but relatively wide, indicating that uncertainty should be incorporated into decision-making for NCVBP volume forecasting rather than relying solely on point estimates. In summary, the proposed TBATS-XGB hybrid model informs NCVBP drug volume planning and addresses the limitations of conventional empirical estimation methods, which are ill-equipped to handle structural demand shifts and seasonal fluctuations.

## Data Availability

The raw data supporting the conclusions of this article will be made available by the authors, without undue reservation.
